# Parvovirus 4–like Virus in Blood Products

**DOI:** 10.3201/eid1603.090746

**Published:** 2010-03

**Authors:** Jozsef Szelei, Kaiyu Liu, Yi Li, Sandra Fernandes, Peter Tijssen

**Affiliations:** Institut National de la Recherche Scientifique–Institut Armand-Frappier, Laval, Quebec, Canada (J. Szelei, K. Liu, Yi Li, S. Fernandes, P. Tijssen); Central People’s Republic of China Normal University, Wuhan, People’s Republic of China (Y. Li)

**Keywords:** Parvovirus infections, factor VIII, plasma, hemophilia, pharmaceutical preparations, capsid protein VP1, NS1 protein, viruses, dispatch

## Abstract

Porcine plasma and factor VIII preparations were screened for parvovirus 4 (PARV)–like viruses. Although the prevalence of PARV4-like viruses in plasma samples was relatively low, viruses appeared to be concentrated during manufacture of factor VIII. PARV4-like viruses from human and porcine origins coevolved likewise with their hosts.

In 2005, a previously unknown virus, parvovirus 4 (PARV4), was detected in a plasma sample from a hepatitis B–positive injection drug user (IDU) (*1*). Although PARV4 was subsequently detected in plasma from healthy donors, its prevalence is higher in samples from IDUs, AIDS patients, and hepatitis C virus–infected persons (*2*,*3*). In recent serologic studies, 67% of HIV-infected IDUs had antibodies to PARV4, whereas non-IDU controls were seronegative (*4*) This increased prevalence in IDUs and persons with hemophilia most likely reflects parenteral transmission of the virus (*4*,*5*). Furthermore, PARV4 was frequently detected in human coagulation factor concentrates prepared from older plasma samples (*6*). The lower detection frequency in current concentrates may be due to exclusion of high-risk batches, e.g., from IDU or hepatitis C virus–infected persons during plasma collection, and to improved purification methods. The presence of PARV4 in plasma suggests a viremic phase enabling spread of the virus to different organs. Even though recent studies by Kleinman et al. indicate that parvovirus B19 is not readily transmitted to susceptible hosts by blood component transfusion, similar evaluation of PARV4 transmission will be invaluable in assessing the need to routinely screen for this emerging virus (*7*).

PARV4 contains a 5-kb single-stranded DNA genome with inverted terminal repeats  and a large open reading frame (ORF) in each half of the genome coding for nonstructural protein (NS) and structural protein, respectively. PARV4-like viruses form a separate cluster among the parvoviruses (*1*,*8*). Three genotypes of human PARV4 parvoviruses with ≈93% nucleotide sequence identity have been described. The sequence of genotype 1 (PARV4-g1) is highly conserved, whereas that of genotype 2 (PARV4-g2 [formerly PARV5]) is somewhat more diverse. PARV4-g2 is found mostly in older coagulation factor concentrates (1960s–1980s), suggesting that genotype 1 emerged recently (*6*,*8*). A third genotype (PARV4-g3) was isolated from persons in sub-Saharan Africa (*9*). Additionally, PARV4-like viruses with a 60%–65% nucleotide identity were recently identified at high frequencies in porcine and bovine tissue samples in People’s Republic of China (*10*).

In this study, porcine plasma samples and factor VIII (FVIII) concentrates used by persons with hemophilia who have autoimmune antibodies against human FVIII were investigated for PARV4-like viruses. We then determined the degree of identity of these isolates with the human virus.

## The Study

Plasma samples from healthy pigs were collected in Great Britain in 2001. Initially, these samples were tested for PARV4-like viruses by using previously described degenerate PCR primers (*10*). DNA was extracted from samples by using the High Pure DNA Isolation Kit (Roche Applied Science, Roche Diagnostics Canada; Laval, Quebec, Canada). Only 3 of the 98 plasma samples contained detectable amounts of PARV4-like viruses. To further study these porcine viruses, we obtained nearly full-length genomes from overlapping PCR fragments. Primers designed for these PCRs were PrS1: 5′-CCACACCTACCTCGCCTATAAGAATCAG-3′; PrAS1: 5′-CTCCACTTGTTCAGCACGGGATCC-3′; PrS2: 5′-CCACGAGCTGGAAGTCTTTA-3′; PrAS2: 5′-GGAGTCCGCATACCCATAACAGGCTG-3′; PrS3: 5′-GTGTACCGCAGTGGGAGCCATG-3′; and PrAS3: 5′-TTCTGGGCAACCCACTGATCAGAAGG-3′. The nearly full-length clones were sequenced by primer-walking. Genomic analysis confirmed that these viruses were related to the PARV4 viruses and were close relatives of the recently identified porcine hokoviruses (PHoVs) (*10*).

**Figure 1:**
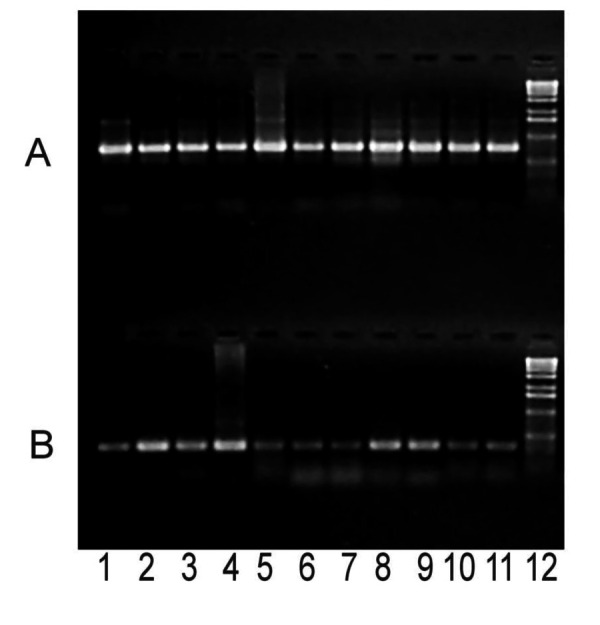
Parallel PCR amplification of PARV4-like (A) and PPV (B) by using purified DNA from clotting FVIII preparations. The results of this PCR usually suggested a higher PARV4 load despite the higher efficiency of the PPV PCR (J. Szelei and P. Tijssen, unpub. data). This finding was confirmed with the quantitative MIMIC PCR method for PPV (*11*). Numbers indicate different lots of FVIII prepared in 1:1994A, 2:1994B, 3:1996A, 4:1996B, 5:1999, 6:2000A, 7:2000B, 8:2001A, 9:2001B, 10:2001C, 11:2001D, and 12: DNA marker (1-kb ladder; Invitrogen, Carlsbad, CA USA). PARV4, parvovirus 4; PPV, porcine parvovirus; FVIII, factor VIII.

We also confirmed the moderate frequency of PARV4-like viremia in the previously tested pig plasma samples with a more sensitive PCR assay by using specific primers PrS4 (5′-AGTTACGGGGGACCGCTACAGTG-3′) and PrAS3. In contrast, examination of 11 commercial clotting FVIII preparations showed that all of these independent lots contained substantial amounts of PARV4-like parvovirus, whereas the level of porcine parvovirus DNA was generally lower in the corresponding samples (Figure 1). Similar to the plasma samples, long overlapping PCR fragments were amplified from the FVIII preparations to obtain nearly full-length sequences. Their analysis provided information about the evolution of PARV4-like viruses, during nearly a decade, in pigs. Sequence data were registered by GenBank (accession nos. Cl2001A: FJ982246; Cl2001B: FJ982247; Cl2001C: FJ982248; F8–1994A: FJ982249; F8–1994B: FJ982250; F8–1996A: FJ982251; F8–1996B: FJ982252; F8–1999: FJ982253; F8–2000A: FJ982254; and F8–2000B: FJ982255). Phylogenetic and molecular evolutionary analyses were conducted by using MEGA version 4 (*12*).

**Figure 2:**
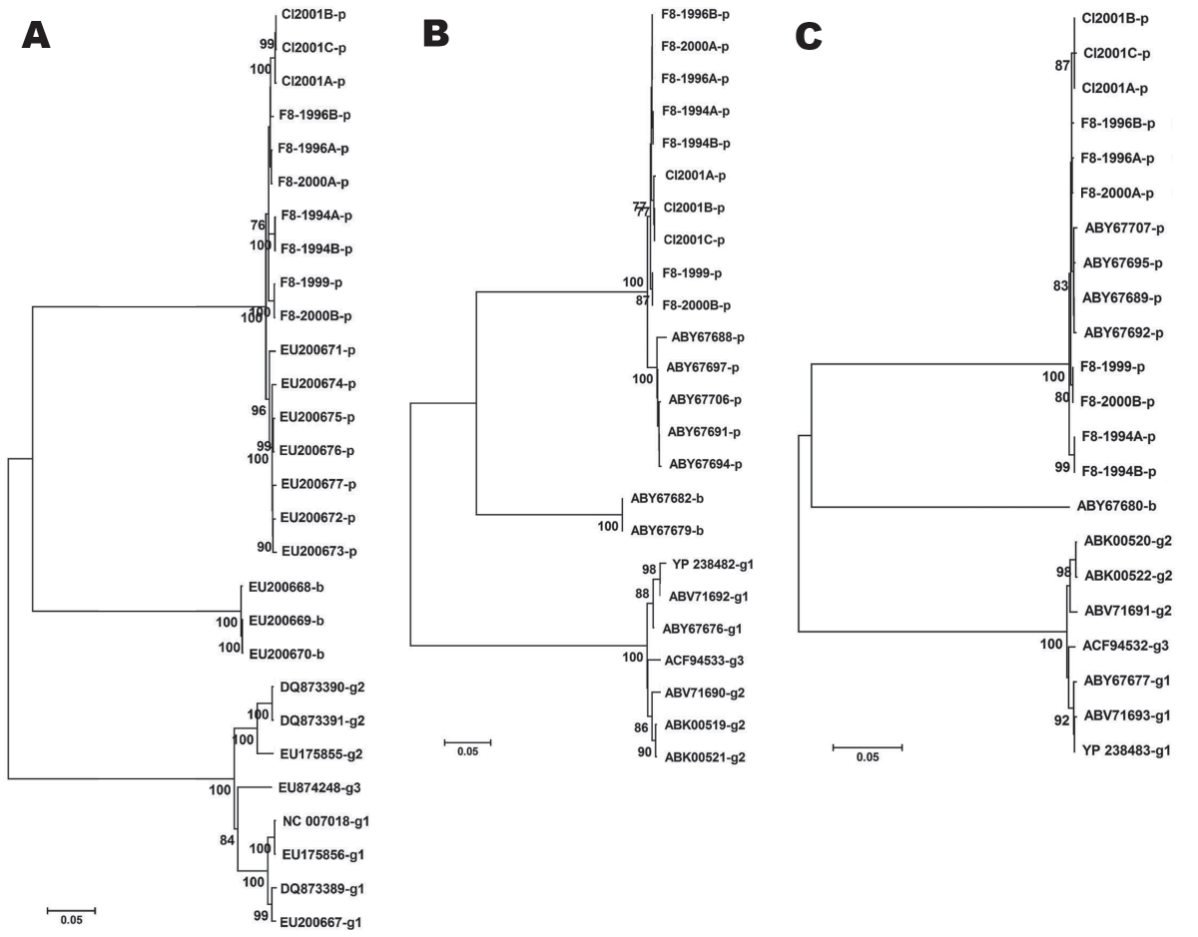
Construction of phylogenic trees for newly identified porcine viruses and comparison with previously identified prototype parvovirus 4 (PARV4)–like sequences. Sequences of other PARV4-like viruses indicated by the accession numbers were obtained from GenBank, and their origins are marked by letters (p, porcine; b, bovine; PARV4-g1, g2, g3, human parvovirus 4 genotypes 1, 2, and 3). ClustalW-aligned genomes (A) and nonstructural (NS) protein (B) and viral protein (VP) (C) were all trimmed to obtain sequences with similar lengths. All computer analysis was performed by using the neighbor-joining method. Branches corresponding to partitions reproduced in <70% bootstrap replicates are collapsed. The tree is drawn to scale, and the percentage of replicate trees in which the associated taxa clustered together in the bootstrap test (1,000 replicates) are shown below the branches. F8-year, year of the factor VIII lot; Cl-year, plasma samples and year of collection. Scale bar represents the number of nucleotide (A) or amino acid (B, C) substitutions per site.

The genomes of these newly isolated PARV4-like viruses were similar to the PHoVs previously identified in Hong Kong Special Administrative Region, People’s Republic of China. Although, these new isolates showed some diversity (98%–99% identity), they differed somewhat more from the PHoVs (97%–98% identity). The viral protein (VP)-ORF was highly conserved (99%), whereas the NS-ORF showed more diversity (97%–98%). Genomic and protein-coding sequences were also compared with other PARV4-like viruses (Tables 1, 2). Phylogenic analysis using neighbor-joining and maximum parsimony methods demonstrated that PHoVs grouped together, whereas PARV4-like sequences from FVIII prepared at different times were less uniform (Figure 2). Older FVIII PARV4 contaminants (especially from 1994) were related more closely to the bovine hokoviruses (BHoVs) and to PARV4-g2. Finally, analysis of the newly identified virus genomes showed an alternative coding sequence inside of the VP gene with a recognizable relationship to small alternatively translated (SAT) proteins (*13*). In the porcine PARV4-like viruses, the start codon for the SAT protein was 3 nt downstream relative to the position of SAT-ATG in the human and bovine PARV4 viruses. Although the SAT protein was 67 aa in all the characterized human PARV4 viruses, porcine and bovine PARV-like viruses contained SAT proteins with 84 aa. The amino acid sequences of the SAT proteins were highly conserved in each PARV4 virus group; however, they differed greatly between PARV4 viruses belonging to different host species (Table 2).

**Table 1:** Percentage diversity of genome sequences of PARV4-like viruses*†

Genotype	PARV4-p	PHoV	BHoV	PARV4-g1	PARV4-g2
PARV4-p	98–99				
PHoV	97–98	98–99			
BHoV	62	62	99		
PARV4-g1	58	58	60	98–100	
PARV4-g2	58–59	58	59–60	91–92	96–99
PARV4-g3	58	58	60	92	91–92

*PARV4, parvovirus type 4; PARV4-p, porcine PARV-4; PHoV, porcine hokovirus; BHoV, bovine hokovirus; PARV4-g1, PARV4 genotype 1; PARV4-g2, PARV4 genotype 2; PARV4-g3, PARV4 genotype 3. †Pairwise sequence comparisons were performed by using the ClustalW program (www.ebi.ac.uk/Tools/clustalw) as described in Figure 2 and percentages of sequence identities were calculated. Nucleotide sequences representing the equivalent regions (position 248–5088, numbered according to the PARV4 sequence NC_007018) were used to align the DNA fragments.

**Table 2:** Analysis of relationships among the protein sequences of PARV4-like viruses*†

Sequence	PARV4-p	PHoV	BHoV	PARV4-g1	PARV4-g2	PARV4-g3
PARV4-p						
NS	99–100	(99)	(80)	(68)	(68)	(68)
VP	99–100	(99)	(79)	(77)	(78)	(77)
SAT	100					
PHoV						
NS	97–98	98–99	(79)	(68)	(68)	(68)
VP	99	99	(79)	(77)	(77)	(77)
SAT	98–100	98–100				
BHoV						
NS	67–68	67	99	(70)	(70)	(70)
VP	66	66	NA	(78)	(78)	(78)
SAT	79	79	100			
PARV4-g1						
NS	53–55	53–54	56–57	96–99	(99)	(98)
VP	65	65	65	99	(99)	(98–99)
SAT	59	59	59	100		
PARV4-g2						
NS	54–55	53–54	56	96–97	98–99	(98)
VP	65	65	64–65	98	98–99	(98)
SAT	59	59	59	100	100	
PARV4-g3						
NS	54	53–54	56	96–97	96–97	NA
VP	65	65	64	98	97–98	NA
SAT	59	59	59	100	100	

*PARV4, parvovirus type 4; PARV4-p, porcine PARV-4; PHoV, porcine hokovirus; BHoV, bovine hokovirus; PARV4-g1, PARV4 genotype 1; PARV4-g2, PARV4 genotype 2; PARV4-g3, PARV4 genotype 3; NS, nonstructural protein; VP, viral protein; NA, no alignment; SAT, small alternatively translated proteins. †Numbers indicate percentages of amino acid sequence identity; numbers in parentheses indicate percentages of amino acid similarity (preserved physicochemical properties). Sequence similarity was not calculated for the SAT proteins, because of their relatively smaller size. When only 1 sequence was available (e.g., VP of BHoV), no alignment was performed.

## Conclusions

Improved virus detection methods have facilitated the discovery of new viruses and have provided insight into the existence of a wide variety of potentially pathogenic strains in biopharmaceutical products. Plasma samples, collected from individual pigs in 2001–2002, and FVIII samples, prepared during 1994–2001, were tested for PARV4-like viruses.

Sequence analysis showed that PARV4-like viruses may have undergone some degree of selective pressure during this time because the genomes sequenced showed a greater variability than the porcine parvovirus NS sequences isolated from the same samples (J. Szelei and P. Tijssen, unpub. data). In the current study, comparison of the genomic and NS protein coding sequences indicated that viruses in the older samples were more closely related to BHoV and PARV4-g2 (Figure 2). Fewer changes were observed in the VP coding sequence (Table 2). Because VPs are responsible for the entry of parvoviruses, they usually adapt to host-specific receptor(s). The presence of PARV4-g2–like isolates in older samples and the omnipresence of PARV4-like viruses in more recent samples suggested that the porcine PARV4-like virus and human PARV4 may have similarly evolved (*8*). These new parvovirus isolates from Great Britain would belong to a different cluster of porcine PARV4-like viruses than the hokoviruses from Hong Kong Special Administrative Region.

Although older isolates shared more identity with BHoV and PARV4-g2, the substantial differences in the DNA sequences of PARV4-like viruses from different species (human, bovine, pig) suggested that they would have diverged a long time ago. This hypothesis was also supported by the sequence stabilization of the SAT proteins, which may play important host-specific roles in the viral exit (*13*). Nevertheless, the existence of a wide variety of different PARV4 strains, most of which result in chronic infections, could provide a basis for an evolutionary jump and recombination and should raise major concerns about the dangers of parenteral transmission.

## References

[1] Jones MS, Kapoor A, Lukashov VV, Simmonds P, Hecht F, Delwart E. New DNA viruses identified in patients with acute viral infection syndrome. J Virol. 2005;79:8230–6. 10.1128/JVI.79.13.8230-8236.2005 15956568 PMC1143717

[2] Fryer JF, Delwart E, Bernardin F, Tuke PW, Lukashov VV, Baylis SA. Analysis of two human parvovirus PARV4 genotypes identified in human plasma for fractionation. J Gen Virol. 2007;88:2162–7. 10.1099/vir.0.82620-0 17622618

[3] Longhi E, Bestetti G, Acquaviva V, Foschi A, Piolini R, Meroni L, Human parvovirus 4 in the bone marrow of Italian patients with AIDS. AIDS. 2007;21:1481–3. 10.1097/QAD.0b013e3281e38558 17589196

[4] Sharp CP, Lail A, Donfield S, Simmons R, Leen C, Klenerman P, High frequencies of exposure to the novel human parvovirus PARV4 in hemophiliacs and injection drug users, as detected by a serological assay for PARV4 antibodies. J Infect Dis. 2009;200:1119–25. 10.1086/605646 19691429 PMC2914696

[5] Simmonds P, Manning A, Kenneil R, Carnie FW, Bell JE. Parenteral transmission of the novel human parvovirus PARV4. Emerg Infect Dis. 2007;13:1386–8. 18252117 10.3201/eid1309.070428 PMC2857296

[6] Schneider B, Fryer JF, Oldenburg J, Brackmann HH, Baylis SA, Eis-Hübinger AM. Frequency of contamination of coagulation factor concentrates with novel human parvovirus PARV4. Haemophilia. 2008;14:978–86. 10.1111/j.1365-2516.2008.01800.x 18565125

[7] Kleinman SH, Glynn SA, Lee TH, Tobler LH, Schlumpf KS, Todd DS, A linked donor-recipient study to evaluate parvovirus B19 transmission by blood component transfusion. Blood. 2009;114:3677–83. 10.1182/blood-2009-06-225706 19687508 PMC2766683

[8] Manning A, Willey SJ, Bell JE, Simmonds P. Comparison of tissue distribution, persistence, and molecular epidemiology of parvovirus B19 and novel human parvoviruses PARV4 and human bocavirus. J Infect Dis. 2007;195:1345–52. 10.1086/513280 17397006 PMC7109978

[9] Simmonds P, Douglas J, Bestetti G, Longhi E, Antinori S, Parravicini C, A third genotype of the human parvovirus PARV4 in sub-Saharan Africa. J Gen Virol. 2008;89:2299–302. 10.1099/vir.0.2008/001180-0 18753240

[10] Lau SK, Woo PC, Tse H, Fu CT, Au WK, Chen XC, Identification of novel porcine and bovine parvoviruses closely related to human parvovirus 4. J Gen Virol. 2008;89:1840–8. 10.1099/vir.0.2008/000380-0 18632954

[11] Zádori Z, Szelei J, Lacoste MC, Li Y, Gariépy S, Raymond P, A viral phospholipase A2 is required for parvovirus infectivity. Dev Cell. 2001;1:291–302. 10.1016/S1534-5807(01)00031-4 11702787

[12] Tamura K, Dudley J, Nei M, Kumar S. MEGA4: Molecular evolutionary genetics analysis (MEGA) software version 4.0. Mol Biol Evol. 2007;24:1596–9. 10.1093/molbev/msm092 17488738

[13] Zádori Z, Szelei J, Tijssen P. SAT: a late NS protein of porcine parvovirus. J Virol. 2005;79:13129–38. 10.1128/JVI.79.20.13129-13138.2005 16189014 PMC1235825

